# Pirfenidone inhibits transforming growth factor-β1-induced fibrogenesis by blocking nuclear translocation of Smads in human retinal pigment epithelial cell line ARPE-19

**Published:** 2012-04-21

**Authors:** Kyungsun Choi, Kihwang Lee, Seung-Wook Ryu, Minju Im, Koung Hoon Kook, Chulhee Choi

**Affiliations:** 1 Department of Bio and Brain Engineering, KAIST, Daejeon, Republic of Korea; 2 Department of Ophthalmology, School of Medicine, Ajou University, Suwon, Republic of Korea

## Abstract

**Purpose:**

Transforming growth factor-β (TGF-β) plays a key role in transforming retinal pigment epithelial (RPE) cells into mesenchymal fibroblastic cells, which are implicated in proliferative vitreoretinopathy. Herein, we tested the effect of pirfenidone, a novel antifibrotic agent, on TGF-β1-mediated fibrogenesis in the human RPE cell line ARPE-19.

**Methods:**

The effect of pirfenidone on the TGF-β1-induced phenotype in ARPE-19 cells was measured with immunocytochemistry as the change in F-actin. Fibronectin and collagen production was measured with enzyme-linked immunosorbent assay, and cell migration activity was investigated using a scratch assay. Immunoblot analyses of cofilin, sma and mad protein (smad) 2/3, p38 mitogen-activated protein kinase, c-Jun N-terminal kinase, and extracellular signal-related kinase expression were conducted to elucidate the cell signaling networks that contribute to the antifibrotic effect of pirfenidone.

**Results:**

Treatment with TGF-β1 induced typical phenotypic changes such as formation of stress fiber running parallel to the long axis of cells and enhanced migration and production of extracellular matrix components such as collagen type I and fibronectin. This fibroblast-like phenotype induced by TGF-β1 was significantly inhibited by pretreatment with pirfenidone in a dose-dependent manner. We also elucidated the TGF-β signaling pathways as the target of the inhibitory effect of pirfenidone. Pirfenidone inhibited TGF-β signaling by preventing nuclear accumulation of active Smad2/3 complexes rather than phosphorylation of Smad2/3.

**Conclusions:**

These results collectively provide a rational background for future evaluation of pirfenidone as a potential antifibrotic agent for treating proliferative vitreoretinopathy and other fibrotic retinal disorders.

## Introduction

Fibrotic diseases of the posterior segment of the eye include proliferative vitreoretinopathy, retinopathy of prematurity, diabetic retinopathy, and age-related macular degeneration. Retinal pigment epithelial (RPE) cells, which are normally located in the external cell layer of the retina, are the most critical contributors to the development of fibrotic diseases of the eye [1,2]. Hypoxia, inflammation, and mechanical insults cause RPE cells to undergo a transformation into fibroblast-like cells via a process known as the epithelial-to-mesenchymal transition (EMT) [3-5]. This mesenchymal transition also contributes to wound healing, tissue regeneration, and organ fibrosis after tissue injury. At sites of chronic inflammation, organ-composed cells such as the alveolar epithelial cell, hepatocyte, and tubular epithelial cell, undergo the EMT and then transform into fibroblasts. Accumulated fibroblasts produce excess collagen and other matrix components leading to scar tissue formation and progressive tissue injury of the heart, lung, liver, and kidney [6-8].

In the process of converting from an epithelial cell into a mesenchymal cell, the cells lose epithelial characteristics such as polarity and specialized cell-to-cell contact, and acquire migratory mesenchymal properties. These processes are mediated by expression of cell surface molecules, cytoskeletal reorganization, and extracellular matrix (ECM) components and activation of transcription factors [9,10]. In particular, transforming growth factor-β (TGF-β) signaling has been considered a key effector of the EMT, and is known to induce the transformation of RPE cells into fibroblast-like cells in vitro [11-13], suggesting that targeting TGF-β signaling provides new insights for developing novel therapeutic interventions [14,15].

Pirfenidone (5-methyl-1-phenyl-2-[1H]-pyridone), a small compound with combined anti-inflammatory and antioxidative action, is also known for its antifibrotic action in experimental animal models of lung, kidney, and liver fibrosis [16-19]. Clinical trials have shown the effectiveness of pirfenidone in extending survival time and improving pulmonary function in patients with idiopathic pulmonary fibrosis [20,21]. We have previously shown that non-toxic concentrations of pirfenidone have significant antifibrotic effects on orbital fibroblasts from patients with thyroid-associated ophthalmopathy [22]. Surprisingly, the molecular mechanisms responsible for the antifibrotic action of pirfenidone have not yet been determined. In this study, we investigated the molecular mechanisms of pirfenidone for the inhibitory action for TGF-β1-induced fibrogenesis in ARPE-19 cells.

## Methods

### Cell culture and reagents

Human retinal pigment epithelial cell line (ARPE-19) cells obtained from the American Type Cell Culture (ATCC, Manass, VA) [23] were maintained in Dulbecco’s modified Eagle’s minimum essential medium (DMEM)/F-12 medium (1:1 mixture of DMEM and Hank’s balanced salt solution [HBSS]; Grand Island, NY) supplemented with 10% fetal bovine serum (FBS), 100 U/ml penicillin G, 100 mg/l streptomycin, and 2 mmol/l L-glutamine in a humidified incubator at 37 °C under 5% CO_2_ in 95% air as described previously [24]. Pirfenidone was purchased from Sigma (St. Louis, MO). Human recombinant TGF-β1 was purchased from R&D Systems (Minneapolis, MN). Specific pharmacological inhibitors of p38 mitogen-activated protein kinase (MAPK, SB202190) and Rho (hydroxyfasudil) were obtained from Calbiochem (La Jolla, CA). Antibodies specific to β-actin, N-cadherin, cofilin, phospho-cofilin (Ser^3^), sma and mad protein (smad) 2/3, phospho-Smad2/3 (Ser^465/467^), p38 mitogen-activated protein kinase (MAPK), phospho-p38 (Thr^180^/Tyr^182^), c-Jun N-terminal kinase (JNK), phosphor-JNK (Thr^183^/Tyr^185^), extracellular signal-related kinase (Erk)1/2, phosphor-Erk1/2 (Thr^202^/Tyr^204^), poly (ADP-ribose) polymerase (PARP), and α-tublin were purchased from Cell Signaling (Beverly, MA). Rhodamine-labeled phalloidin and propidium iodide were purchased from Molecular Probes (Eugene, OR).

### Enzyme-linked immunosorbent assay

ARPE-19 cells were incubated in the absence or presence of pirfenidone for 1 h and then treated with TGF-β1 (10 μg/l) for an additional 48 h. All of the cultures contained the same concentration of dimethyl sulfoxide. The supernatants were processed for collagen type I C-terminal peptide and fibronectin enzyme-linked immunosorbent assay (ELISA) kits (Takara, Tokyo, Japan) according to the protocol provided by the manufacturer. The color reaction was measured at 450 nm. Collagen type I C-terminal peptide and fibronectin protein values were normalized by the protein concentration of the total cell lysates.

### Immunocytochemistry

ARPE-19 cells were cultured in four-well multichamber and then supplemented with TGF-β1 (10 μg/l) for 48 h in the absence or presence of pirfenidone or hydroxyfasudil. Next, the cells were rinsed for 3 min in 1× phosphate buffered saline (PBS, 137 mmol/l NaCl, 2.7 mmol/l KCl, 10 mmol/l Na_2_HPO_4_, 2 mmol/l KH_2_PO_4_, pH 7.4), fixed in 5% paraformaldehyde for 30 min, and permeabilized with 0.2% Triton (Sigma, Calbiochem, CA) in PBS for 20 min. The cells were then incubated for 1 h with rhodamine-labeled phalloidin (diluted 1:100). After being washed with PBS, the cells were mounted with FluorSave reagent (Calbiochem) and analyzed with confocal microscopy (Carl Zeiss, Gottingen, Germany).

### Cell migration assay

Cell migration was evaluated by assaying the closure of a liner defect produced in a cell monolayer culture as described previously [25]. The defect was generated in a confluent culture of ARPE-19 cells by scraping with a micropipette tip. The cells were treated with TGF-β1 in the absence or presence of various pharmacological inhibitors. After 48 h, the cells were analyzed with phase contrast microscopy. Migration distance was determined using i-Solution (iMTechnology, Seoul, Korea), and the shortest distance between the cells that had moved into the wounded region and their respective starting points was determined.

### Immunoblot analysis

Cell lysates were subjected to sodium dodecyl sulfate–PAGE, then transferred to nitrocellulose, and probed with antibodies. The blots were developed using chemiluminescence (AbFrontier, Seoul, Korea). To investigate the nucleocytoplasmic shuttling of Smads, the nuclear extract was separated from the cytoplasmic fraction using a Nuclear/Cytosol Fractionation Kit (BioVision, Mountain View, CA) according to the manufacturer’s protocol.

### Statistical analysis

The data are presented as the mean±SD. The level of significance for comparisons between samples was determined with one-way ANOVA with Tukey’s honest significant difference post-hoc test using InStat software (GraphPad Software Inc., San Diego, CA).

## Results

### Pirfenidone inhibits transforming growth factor-β1 induced fibroblastic phenotypes in ARPE-19 cells

**Figure 1:**
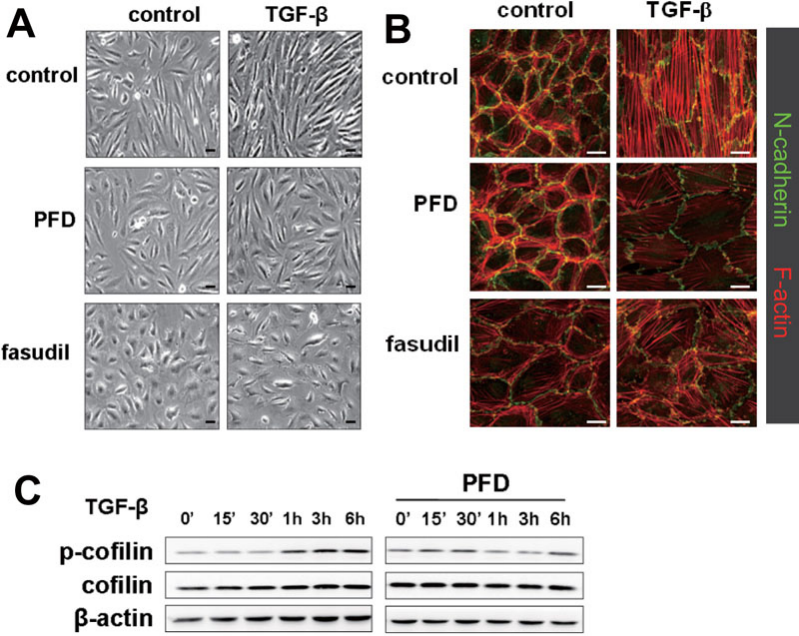
Pirfenidone inhibited transforming growth factor- β1 (TGF- β1)-induced morphological changes and actin rearrangement in a human retinal pigment epithelial cells, ARPE-19. **A**: ARPE-19 cells were incubated in the absence or presence of pirfenidone (500 mg/l) or hydroxyfasudil (10 μmol/l) for 1 h, treated with TGF-β1 (10 μg/l) for an additional 48 h, and visualized with phase contrast microscopy. The data shown are representative of at least four independent experiments. Magnification, 100×. Scale bar=20 μm. **B**: Cells were incubated in the absence or presence of pirfenidone (500 mg/l) or hydroxyfasudil (10 μmol/l) for 1 h, treated with TGF-β1 (10 μg/l) for an additional 48 h, and stained with rhodamine-labeled phalloidin for F-actin and fluorescein isothiocyanate (FITC)-conjugated antibodies for N-cadherin. The data shown are representative of at least three independent experiments. Magnification, 400×. Scale bar=20 μm. **C**: Cells were incubated in the absence or presence of pirfenidone (500 mg/l) for 1 h and then treated with TGF-β1 (10 μg/l) for varying time periods. The total cell lysates were subjected to immunoblot analysis for phospho-cofilin, cofilin, and β-actin. The data shown are representative of at least two independent experiments. Control: untreated, PFD: pirfenidone, fasudil: hydroxyfasudil.

**Figure 2:**
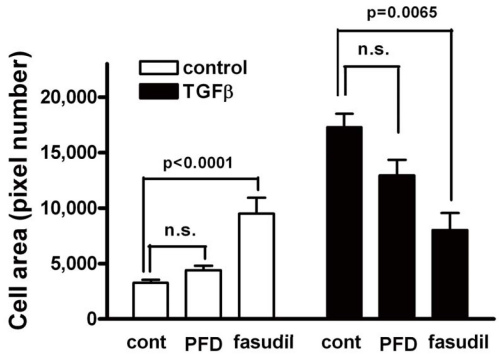
Pirfenidone had little effect on transforming growth factor (TGF)-β1-induced increase in the cell surface area. Cells were incubated in the absence or presence of pirfenidone (PFD, 500 mg/l) or hydroxyfasudil (10 μmol/l) for 1 h, treated with transforming growth factor- β1 (TGF)-β1 (10 μg/l) for an additional 48 h, and stained with fluorescein isothiocyanate (FITC)-conjugated antibodies for N-cadherin. Cell size was measured as the number of pixels in the cell boundary indicated by N-cadherin staining.

To investigate the effect of pirfenidone on the TGF-β1-induced EMT, we first examined whether the TGF-β1-induced morphological changes were affected by pirfenidone. Treatment with TGF-β1 induced prominent morphological changes in ARPE-19 cells, including elongated and spindle-like shapes, which were noticeably suppressed by pretreatment with pirfenidone or hydroxyfasudil, a Rho kinase inhibitor (Figure 1A). Next, we examined cytoskeletal reorganization by staining for F-actin in response to TGF-β1. As the cells began to form spindle-like processes upon TGF-β1 stimulation, the distribution of F-actin was arrayed in a series of linear and parallel stress fiber-like structures. Stress fiber formation was severely disorganized and failed to develop into more mature and spindle-like structures in the presence of pirfenidone or hydroxyfasudil (Figure 1B). Cells treated with TGF-β1 exhibited up to a fivefold increase in cell surface area compared to unstimulated control cells, which is consistent with a previous report [26]. Treatment with hydroxyfasudil alone increased cell surface area and inhibited the TGF-β1-induced increase in the cell surface area; while pirfenidone had little effect on cell size (Figure 2).

Cofilin, a small actin-binding protein, is involved in cell mobility and invasion via controlling actin polymerization [27]. Phosphorylation of cofilin is responsible for TGF-β1-induced actin polymerization, which can be blocked by pretreatment with chemical inhibitors of RhoA or Rho kinase (ROCK) [28]. To determine the inhibitory effects of pirfenidone on a downstream effector of RhoA, we analyzed the phosphorylation of cofilin at serine 3 in ARPE-19 cells with immunoblot analysis. As expected, preincubation with pirfenidone suppressed the TGF-β1-induced phosphorylation of cofilin (Figure 1C). These results collectively indicate that TGF-β1-induced actin rearrangements and morphological changes are mediated by the RhoA pathway and these events are significantly suppressed by pirfenidone.

### Pirfenidone suppresses the transforming growth factor-β1-induced expression of extracellular matrix components in ARPE-19 cells

**Figure 3:**
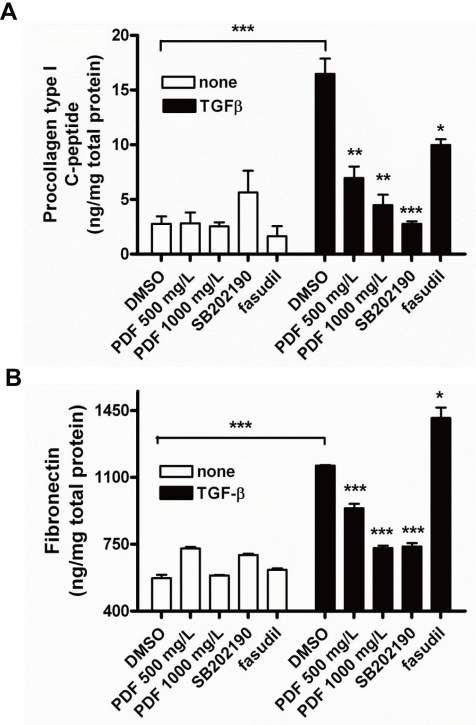
Pirfenidone inhibited transforming growth factor- β1 (TGF-β1)-induced expression of extracellular matrix. Components in human retinal pigment epithelial cells (ARPE-19) cells. Cells were incubated in the absence or presence of SB202190 (10 μmol/l), hydroxyfasudil (10 μmol/l), or varying doses of pirfenidone for 1 h and then treated with TGF-β1 (10 μg/l) for an additional 48 h. The supernatants were assayed with ELISA for the level of collagen type I (**A**) or fibronectin (**B**). Control: untreated, PFD: pirfenidone, fasudil: hydroxyfasudil. Samples significantly different from the control sample treated with DMSO were indicated with symbols (*, n=5, error bar indicates standard deviation [SD], p<0.05; **, p<0.01; ***, p<0.001).

We analyzed the effect of pirfenidone on the basal and TGF-β1-induced synthesis of collagen type I and fibronectin, the major ECM components of fibrosis. Treatment with TGF-β1 increased the expression of collagen type I by ARPE cells up to eightfold. Since RhoA and p38 MAPK are known to be involved in TGF-β-induced ECM production [24,29], we tested the inhibitory effect of pirfenidone on TGF-β-induced ECM secretion compared with pharmacological inhibitors of RhoA and p38. Pretreatment with pirfenidone, hydroxyfasudil, or SB202190 significantly suppressed the TGF-β1-induced secretion of collagen type I, while the same treatment alone had a minimal effect on the basal level of collagen type I synthesis in ARPE cells (Figure 3A). Parallel results were obtained for fibronectin synthesis; however, hydroxyfasudil had little effect on TGF-β1-induced fibronectin production (Figure 3B). These results are in accordance with previous findings [24,29,30], indicating the differential involvement of RhoA and p38 MAPK pathways in the TGF-β1-induced secretion of the ECM components.

### Pirfenidone abrogates the transforming growth factor-β1-induced migration of ARPE-19 cells

**Figure 4:**
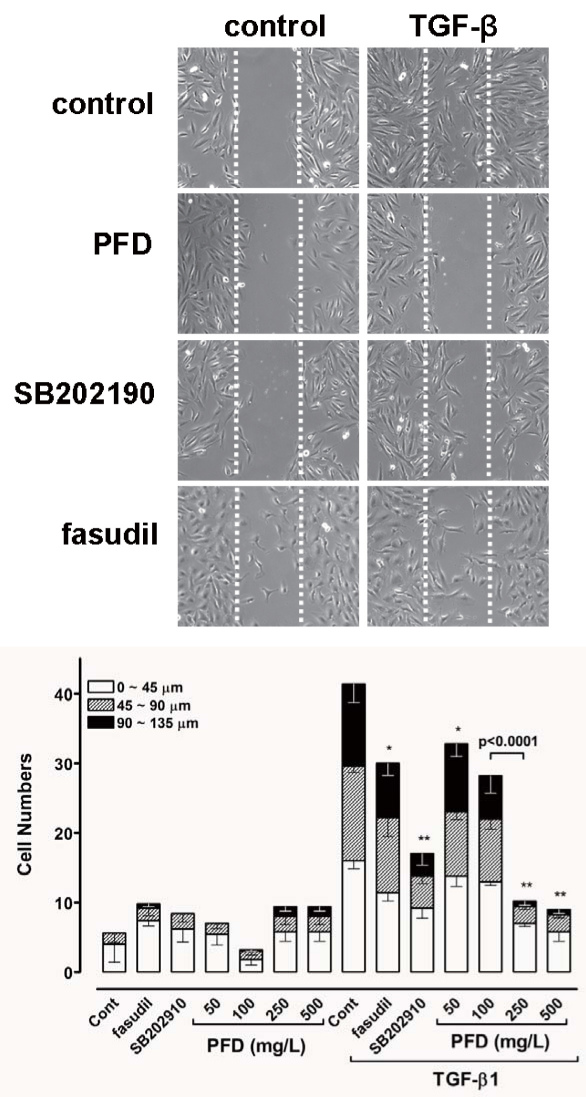
Pirfenidone inhibited transforming growth factor-β1 (TGF-β1)-induced migration of human retinal pigment epithelial cells (ARPE-19) cells. Cells were incubated in the absence or presence of varying doses of pirfenidone, SB202190 (10 μmol/l), or hydroxyfasudil (10 μmol/l) for 1 h and then scratched with a 200-μl micropipette tip to form a cell-free (wounded) area. The cells were then incubated in the absence or presence of TGF-β1 (10 μg/l) for an additional 48 h. The vertical axis represents the number of cells that migrated into the wounded region. Stacked bars display the portion of migratory cells classified by migration distance (the gray bar indicates the number of cells that migrated a moderate distance, and the black bar indicates the number of cells that migrated the longest distance). Samples significantly different from the control sample treated with DMSO were indicated with symbols (*, error bar indicates standard deviation (SD), p<0.05; **, p<0.0001; n=10).

We next tested the effect of pirfenidone on TGF-β1-induced migratory activity, another important phenotype of the EMT. As expected, treatment with TGF-β1 significantly enhanced the migration of cells 48 h after wounding. In contrast, preincubation with pirfenidone, SB202190, or hydrofasudil had significant inhibitory effects on cell migration, and blocked the closure of the defect produced in monolayer cell sheets even in the presence of TGF-β1 (Figure 4). The most significant reduction in motility was noted at pirfenidone concentrations of 250 and 500 mg/l.

### Pirfenidone blocks transforming growth factor-β1-induced nuclear translocation but not phosphorylation of Smads

**Figure 5:**
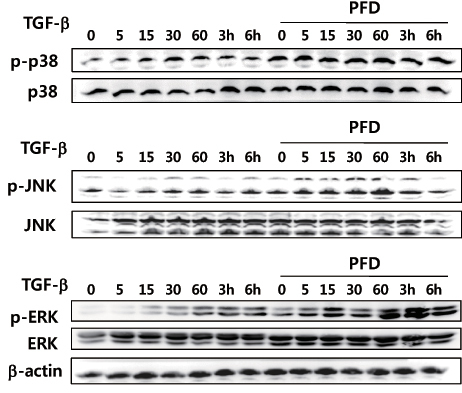
Pirfenidone had no effect on transforming growth factor (TGF)-β1-induced phosphorylation of mitogen-activated protein kinase. Human retinal pigment epithelial cells (ARPE-19) cells were incubated in the absence or presence of pirfenidone (500 mg/l) for 1 h, and then treated with transforming growth factor-β1 (TGF)-β1 (10 μg/l) for varying time periods. Total cell lysates (50 μg total protein) were subjected to immunoblot analysis for p38 mitogen-activated protein kinase, c-Jun N-terminal kinase, extracellular signal-related kinase, and β-actin.

**Figure 6:**
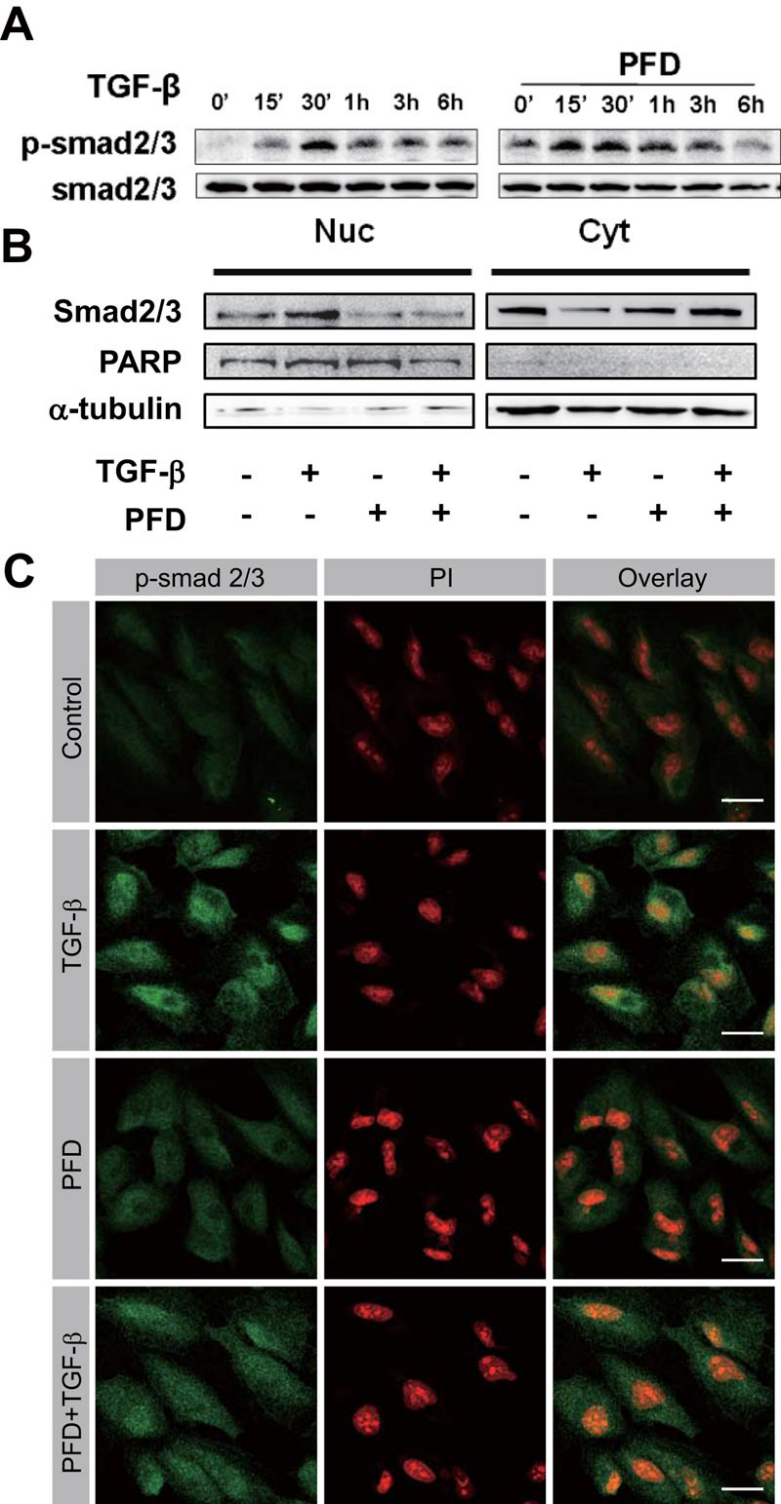
Pirfenidone inhibited transforming growth factor-β1 (TGF)-β1-induced signal transduction. **A**: Human retinal pigment epithelial cells (ARPE-19) cells were incubated in the absence or presence of pirfenidone (500 mg/l) for 1 h, then treated with TGF-β1 (10 μg/l) for varying time periods, and total cell lysates were subjected to immunoblot analysis for phosphor-Smad and Smad2/3. The data shown are representative of three independent experiments. **B**: Nuclear extracts from the cells incubated in the absence or presence of pirfenidone (500 mg/l) for 1 h and then treated with TGF-β1 (10 μg/l) for an additional 30 min were subjected to immunoblot analysis for Smad2/3. Poly (ADP-ribose) polymerase (PARP) was used for a positive control for nuclear compartment; while α-tubulin was used for a positive control for cytosolic fraction. **C**: Cells were incubated in the absence or presence of pirfenidone (500 mg/l) for 1 h, then treated with TGF-β1 (10 μg/l) for 30 min, and stained with antibody against phospho-specific Smad2/3 and secondary antibody conjugated with fluorescein isothiocyanate (FITC; green). Nucleus was counter-stained with propidium iodide (red). Scale bar=20 μm. The data shown are representative of three independent experiments.

Since pirfenidone abrogated TGF-β1-induced EMT-like phenotypic changes, we further investigated the Smad and MAPK signaling pathways responsible for the TGF-β1-induced EMT. Even though MAKPs such as p38, ERK, and JNK were phosphorylated in a time-dependent manner upon TGF-β1 treatment, pretreatment with pirfenidone had no effect on TGF-β1-induced MAPK phosphorylation (Figure 5). TGF-β1 induced time-dependent phosphorylation of Smad2/3 by ARPE-19 cells. Contrary to our expectation, preincubation with pirfenidone had little effect on the TGF-β1-induced phosphorylation of Smad2/3 (Figure 6A). Phosphorylated Smads are known to be translocated into the nucleus for activating or repressing responsible genes [31]. To determine the effect of pirfenidone on the nucleocytoplasmic shuttling of Smads, we performed the immunoblot analysis using nuclear extracts from the cells treated with TGF-β1 in the absence or presence of pirfenidone. Treatment with TGF-β1 induced the nuclear translocation of phosphorylated Smad2/3; while pretreatment with pirfenidone abrogated TGF-β1-induced nuclear localization of the Smads (Figure 6B). The blockage of TGF-β1-induced nuclear translocation of the Smads was confirmed with immunocytochemistry (Figure 6C).

## Discussion

In the present study, we demonstrated the strong inhibitory effect of pirfenidone on the TGF-β1-induced EMT in ARPE-19 cells, based on pirfenidone’s ability to suppress cytoskeletal organization, ECM synthesis, and cellular migration. In addition, we have delineated that the downstream signaling pathways responsible for the TGF-β1-induced EMT, especially the nucleocytoplasmic shuttling of phosphorylated Smads, were blocked by pirfenidone. Although the antifibrotic efficacy of pirfenidone is well established, to the best of our knowledge this is the first study to describe the molecular mechanisms responsible for the biologic activities of pirfenidone in a human RPE cell line.

Pirfenidone exerted its antifibrotic effect through inhibition of heat shock protein 47, a collagen-specific chaperon, resulting in a reduction in collagen synthesis in TGF-β1-induced lung fibroblasts [32]. In animal models of lung fibrosis, pirfenidone also suppressed expression of mRNA and the TGF-β protein [33]. Pirfenidone inhibits platelet-derived growth factor-induced proliferation and collagen production in hepatic stellate cells [34], and reduced expression of procollagen α1 and tissue inhibitors of metalloproteinase-1 through the downregulation of TGF-β1 mRNA in the rat liver fibrosis model [35]. In the renal fibrosis model, pirfenidone was shown to reduce proliferation and activation of renal fibroblasts [36], and prevent expression of collagen and TGF-β [37-39].

The dynamic reorganization of the actin cytoskeleton is tightly regulated by the activation of members of the Rho family of small GTPases, such as the Cdc42/Rac pathway and Rho/ROCK activation [40]. Rac1, Cdc42, and Rho are reciprocally controlled during the formation of lamellipodia, filopodia, and stress fibers, respectively [41,42]. For example, one study found that the inhibition of RhoA induced the expansion of rat mammary adenocarcinoma cells in all directions with the subsequent appearance of round and flat cells due to Cdc24/Rac hyperactivity [41]. The inhibition of Rho or ROCK appears to suppress cell motility in a similar manner, although the phenotypes produced as a result of Rho and ROCK inhibition differ: Rho inhibition led to circumferential expansion under basal conditions, whereas ROCK inhibition resulted in exaggerated growth factor-stimulated expansion [41]. We also observed that unbalanced inhibition of Rho by fasudil had more dramatic effects on cell morphology (see Figure 1B and Figure 2).These findings collectively suggest that pirfenidone might block RhoA and Cdc24/Rac signaling, since treatment of the cells with pirfenidone induced breakdown of stress fibers without affecting cell size. We also confirmed the inhibitory effect of pirfenidone on Rho signaling by showing the suppressive effect of pirfenidone on cofilin phosphorylation, which is known to be mediated by LIM kinase, a well known downstream kinase of Rho signaling.

TGF-β can induce EMT by direct phosphorylation of Smad2/3, or activation of non-Smad signaling pathways including MAP kinase, Rho GTPase, and PI3 kinase-Akt, resulting in repression of epithelial marker genes and activation of mesenchymal markers [43]. Current evidence suggests that the EMT can be therapeutically targeted through disrupting TGF-β signaling at different levels: inhibiting TGF-β expression with RNA interference, antagonizing TGF-β ligand activity, inhibiting TGF-β receptor kinase activity by using small-molecule inhibitors, and intervening in Smad activation [14]. In particular, nuclear translocation of active Smad complexes and subsequent interactions with the general transcription machinery emerged as crucial steps for therapeutic intervention of TGF-β signaling [31]. Here, we demonstrate pirfenidone inhibits TGF-β-activated Smad signaling by preventing nuclear accumulation of phosphorylated Smad2/3, which can suppress Smads signaling without affecting other pathways regulated by TGF-β.

Since the fibrotic transformation of RPE cells is regarded as the main contributor to various fibrotic diseases of the eye [1,2], the inhibitory action of pirfenidone on TGF-β-induced phenotypic changes of a human RPE cell line provides a rationale for a trial of this potential antifibrotic agent in treating proliferative vitreoretinopathy and other fibrotic retinal disorders. However, our results are based on a single human RPE cell line, and further studies involving primary RPE cell cultures are required.
